# Mechanisms, Discovery Strategies, and Emerging Opportunities for AI-Assisted Design of Peptides in Cosmetics and Skin Health

**DOI:** 10.3390/ph19101547

**Published:** 2026-09-30

**Authors:** Jiashu Wang, Mustafa Al-Gafari, Parvin Khosravifar, Thomas D. D. Kazmirchuk, Ashkan Golshani

**Affiliations:** 1 Ottawa Institute of Systems Biology, Faculty of Medicine, University of Ottawa, Ottawa, ON K1H 8M5, Canada; jiashuwang@cmail.carleton.ca (J.W.); mustafaalgafari@cmail.carleton.ca (M.A.-G.); parvinkhosravifar@cmail.carleton.ca (P.K.); tomkazmirchuk@cmail.carleton.ca (T.D.D.K.); 2 Department of Biology, Faculty of Science, Carleton University, Ottawa, ON K1S 5B6, Canada; 3 Institute of Biochemistry, Faculty of Science, Carleton University, Ottawa, ON K1S 5B6, Canada; 4 Designed Biologics Inc., Carleton University, Ottawa, ON K1S 5B6, Canada

**Keywords:** artificial intelligence (AI), AI-guided peptide design, cosmetic peptides, synthetic peptides, peptide discovery, skin health

## Abstract

Synthetic peptides are increasingly used in cosmetic products due to their potential to regulate biological processes related to skin aging, pigmentation, inflammation, extracellular matrix maintenance, and other aspects of skin health. However, the mechanisms of many cosmetic peptides are still not fully understood. Their activities can also be limited by poor stability, skin penetration, off-target effects, and limited clinical evidence. This review discusses the major classes of cosmetic peptides and the molecular mechanisms through which they are proposed to exert their activities. It also examines the main approaches used for peptide discovery. These include discovery from natural and biologically derived sources, library-based screening, rational design, and classical computational methods. More recent artificial intelligence (AI)-guided approaches are then discussed, with particular attention to structure-based and sequence-based peptide design. AI provides new opportunities to search much larger peptide sequence spaces and to prioritize candidates before experimental testing. Structure-based methods can use information about target binding sites to guide peptide design, while sequence-based approaches can identify interaction patterns without requiring detailed 3D structural information. These methods may also help improve peptide specificity, reduce potential off-target interactions, and identify naturally occurring peptides with useful properties. Although the application of AI to cosmetic peptide discovery is still at an early stage, it may provide a useful addition to existing approaches. Future progress will depend on better skin-relevant datasets, improved experimental validation, and design strategies that consider not only target binding, but also selectivity, stability, toxicity, delivery, and formulation compatibility.

## 1. Introduction

Peptides are short chains of amino acids [1]. These amino acids are joined by covalent chemical bonds known as peptide bonds, the same type of bond that links amino acids in proteins in virtually all living organisms [2]. In this respect, peptides and proteins share the same basic chemical structure, although peptides are considerably smaller. A typical protein consists of hundreds to thousands of amino acids, whereas peptides generally contain only a few to a few dozen amino acids. Despite their relatively small size, peptides have diverse biological activities and play important roles in cell signaling, host defense, enzyme regulation, tissue homeostasis and many other processes [3,4].

In recent years, peptides have emerged as an important class of ingredients in cosmetic science. Their growing application reflects both their relative ease of synthesis and their potential to regulate biological processes linked to improved skin appearance and function. In topical formulations, they are designed to improve the appearance of wrinkles, firmness, hydration, and overall skin texture [5,6]. Their proposed utility in cosmetics reflects a broader shift in skin care toward bioactive ingredients. This interest has also encouraged closer examination of the molecular pathways through which cosmetic peptides may influence the biology and health of skin.

Relieving age-related and environmentally induced skin issues is one of the most popular topics in cosmetic sciences. The associated skin changes are related to multiple protein complexes and pathways and their interconnected processes. These include extracellular matrix (ECM) degradation, reduced collagen production, oxidative stress, low-grade inflammation, impaired barrier function, altered intercellular signaling and more [5,6,7]. Peptides have the ability to transport important molecules, to mimic natural signaling and to act on specific biological targets and pathways [8,9,10]. Consequently, they are increasingly considered as important bioactive ingredients. They are categorized mainly into four classes: signaling peptides, carrier peptides, neurotransmitter-inhibiting peptides as well as enzyme-inhibiting peptides [11,12]. They can contribute to skin structure and improve visible skin quality. They do so by stimulating matrix synthesis, modulating neuromuscular signaling, transporting trace elements, or influencing inflammatory and repair-associated processes [13,14]. However, it is important to note that despite their commercial popularity, the mechanisms and degree of efficacy of many cosmetic peptides remain largely unknown. Also, certain peptides that are categorized in one class may play roles in multiple pathways via completely different mechanisms [15,16]. Current studies are limited by the relatively small number of well-controlled experimental groups, differences in experimental methodologies and peptide formulations, the stability of peptides and the ability of peptides to penetrate the stratum corneum [16,17]. While some peptides are supported by in vitro, cell/tissue systems, animal studies or certain clinical findings, others remain supported primarily by proposed mechanisms or even marketing-driven claims [18,19,20]. Translating promising laboratory findings into meaningful clinical benefits remains a major challenge. Many cosmetic peptide studies remain in the early stages [16,17].

Advances in skin biology, peptide engineering, and computational methods are making the rational discovery of cosmetic peptides increasingly feasible [21,22,23,24]. Rather than relying solely on empirical screenings for desired cosmetic effects, it is now reasonable to ask which receptor, enzyme, protein, or molecular interaction should be modulated and then discover/design peptides with that goal in mind. This provides a strong basis for discovering new peptides with more efficient biological activity and enhanced target specificity. In this context, both classical peptide discovery methods and the newer rational computational approaches in peptide engineering are important. Among the latter, artificial intelligence (AI)-guided peptide design offers an innovative path to explore peptide sequence space and prioritize candidates.

This article examines cosmetic peptides from two perspectives: first, their classification and reported molecular mechanisms in skin biology and second, classical and emerging strategies, including AI-guided approaches, for the discovery of next-generation cosmetic peptides. Several previous reviews have summarized the classification, biological activities, delivery, and formulation of cosmetic peptides, for example [10,14]. However, a gap remains in connecting the molecular targets and limitations of existing cosmetic peptides with the strategies used to discover and design new peptide candidates. This gap is particularly apparent for AI-assisted design, where advances in therapeutic peptide research have been rapid, but direct applications to cosmetic peptide discovery remain limited. Therefore, this review places particular emphasis on classical and AI-assisted peptide discovery strategies, their current limitations, and how these approaches may be translated to the development of future cosmetic peptides. Possible future directions in AI-guided peptide discovery are also discussed. This article is a narrative review of representative literature and is not intended as a systematic or exhaustive review.

## 2. Classification of Cosmetic Peptides

These cosmetic peptides are most commonly classified according to their proposed biological role and mechanism of action in skin health [13,14]. A widely used framework in the cosmetic-peptide literature divides them into four major categories: signal peptides, carrier peptides, neurotransmitter-inhibiting peptides, and enzyme-inhibitor peptides. This historical classification is used here as the primary organizational framework, as it remains one of the most commonly applied classifications in the field. It is not intended to be exhaustive. Also, it should be noted that some peptides show overlapping activities and hence cannot be readily assigned to a single category. A summary of the peptides discussed in this review is presented in Table 1 below.

### 2.1. Signal Peptides

Signal peptides are one of the most widely used classes of cosmetic peptides [9]. They mimic endogenous signaling-molecule fragments involved in cellular communication, matrix turnover, or tissue repair. In skin care, they are often associated with stimulation of fibroblast activity and with support of ECM synthesis including collagen-related processes. One of the best studied examples is palmitoyl pentapeptide-4, also known as Pal-KTTKS. It is a fragment of the carboxyl-terminal propeptide of type I collagen that acts as a wound-healing signal [25,49]. Additionally, it has been reported that palmitoyl derivatives can improve skin penetration, so the addition of a palmitoyl group is expected to enhance peptide efficacy [50]. This peptide is thought to promote matrix-related responses in dermal fibroblasts that support the production of collagen and other structural components important for skin firmness and integrity [9]. For this reason, it is incorporated into certain anti-aging formulations intended to improve skin firmness and smoothness, as well as the appearance of fine lines. Peptides in this category are often linked to dermal support and structural maintenance. As a result, they are central to the idea that topical peptides may influence visible signs of skin aging through biologically relevant mechanisms [51].

### 2.2. Carrier Peptides

Carrier peptides usually carry histidine, lysine, glycine, aspartate and glutamate amino acids on their side chains. They presumably function primarily by binding and delivering trace elements, such as copper required for normal skin physiologies, such as wound healing, tissue remodeling, ECM metabolism, UV protection, antioxidant activity, and collagen/elastin stabilization [52,53,54]. Peptide–metal complexes formed in this way are hypothesized to help cells to maintain metal ion solubility and activity while facilitating the delivery to skin cells [55].

The classic example in this group is human peptide glycyl-L-histidyl-L-lysine (GHK), previously known as GHL. This peptide was originally isolated from human plasma and has been investigated as a wound-healing and anti-aging ingredient for many years [56]. It was reported that GHK forms stable coordination complexes with Cu^2+^ (known as GHK-Cu), and was then proposed to facilitate copper transport in skin cells [56]. This form may then deliver Cu^2+^ to the reactions involved in copper-dependent processes such as ECM metabolism. Studies have shown that in the presence of GHK, the copper uptake was increased dramatically in a dose-dependent manner in hepatoma (HTC_4_) cells [57], with further reports of promoted cell growth, collagen production, and the reduction in wrinkles caused by photodamage [27,58,59]. However, the intercellular mechanism of GHK-Cu and its transfer of copper to specific enzymes in human skin remain incompletely characterized.

Similar to GHK-Cu, the complex with manganese (Mn^2+^) has been associated with improvements in photodamage, particularly hyperpigmentation; however, direct intracellular delivery of manganese and engagement in human skin have not been well-demonstrated [60].

### 2.3. Neurotransmitter-Inhibiting Peptides

Unlike signaling or carrier peptides that generally target the dermal fibroblasts and ECM, neurotransmitter-inhibiting peptides are designed to modulate neurotransmitter release at the neuromuscular junction. This kind of inhibition is usually predicted to reduce synaptic vesicle fusion or neuronal excitation, without completely blocking them, hence minimizing the release of acetylcholine to regulate repetitive facial muscle contraction to improve the appearance of wrinkles and expression lines [51,61,62]. Acetyl hexapeptide-8, previously known as acetyl hexapeptide-3 (Argireline^®^), is the best-known representative of this group. This peptide is proposed to interfere with components of the SNARE complex involved in neurotransmitter release [63]. Its exact activity in vivo, however, its extent of penetration into relevant skin sites, and its clinical efficacy remain unclear [16,64].

### 2.4. Enzyme-Inhibitor Peptides

The last group consists of the enzyme-inhibitor peptides. These peptides are proposed to reduce the activity of cosmetically relevant enzymes, including matrix metalloproteinases (MMPs), collagenase, elastase, hyaluronidase and tyrosinase [65,66,67]. These enzymes are mostly involved in processes such as ECM degradation, skin surface remodeling, hydration and pigmentation. One common example is decapeptide-12, a peptide that has been studied primarily in relation to skin pigmentation and tyrosinase-associated pathways [31,32]. Its ability to selectively inhibit tyrosinase is expected to limit the conversion of tyrosine to L-3,4-dihydroxyphenylalanine (L-DOPA), thereby reducing melanin biosynthesis and promoting skin brightening.

This classification provides a practical framework for organizing the diverse peptide ingredients used in cosmetic applications. It should not be viewed as a rigid system. In many cases, peptides are grouped into categories according to their proposed functions rather than detailed molecular mechanisms. In addition, some peptides may exert multiple biological effects depending on concentration and target context. Consequently, this classification is best treated as a useful starting point rather than a complete description of activity. A closer examination of detailed molecular mechanisms for these peptides is needed. This represents a key future direction for research in cosmetic peptides. Understanding of their molecular mechanisms will advance peptide development and support the next generation of cosmetic peptide engineering.

## 3. Molecular Mechanisms

The biological effects attributed to cosmetic peptides are mechanistically diverse and cannot be explained by a single mode of action. Different peptides are thought to modulate different aspects of skin biology. These include regulation of ECM and tissue remodeling, neuromodulatory signaling, inflammation-associated pathways, and pigmentation-related processes [13,14,66]. Although the level of experimental validation and mechanistic support varies among different peptides, these categories still provide a useful framework for discussion. They allow peptide-based cosmetic actives to be examined according to their proposed molecular functions.

### 3.1. Regulation of Extracellular Matrix Synthesis and Dermal Remodeling

One of the most frequently proposed mechanisms for cosmetic peptides is the regulation of ECM synthesis and dermal remodeling [9,18]. The dermal ECM is a complex network of structural proteins and polysaccharides that surrounds dermal cells and provides the skin with strength, elasticity, resilience, and mechanical support [68,69]. During intrinsic aging and photoaging, fibroblast activity declines, collagen and elastin synthesis decreases, and the matrix becomes progressively compromised, leading to wrinkles, skin laxity, and loss of firmness. Several signal peptides used in cosmetic formulations are thought to stimulate dermal fibroblasts to produce ECM components by mimicking endogenous peptide fragments that function in tissue repair or matrix-associated signaling [70,71]. In doing so, they promote fibroblast responses linked to dermal maintenance.

As mentioned above, Pal-KTTKS is one example of such peptides [71,72]. It is derived from a collagen-related peptide motif and is often described as a signal peptide capable of supporting ECM-associated activity. In the cosmetic literature, it has been proposed to stimulate dermal fibroblasts and promote the synthesis of structural macromolecules including collagen and other matrix components to enhance matrix rejuvenation [26,70,73]. Experimental studies support the ability of Pal-KTTKS to increase collagen production in fibroblast systems, but the cell-surface receptor and intracellular signaling cascades responsible for this response remain unidentified [25,26,74].

Another example is palmitoyl tripeptide-1 (Pal-GHK), a derivative of the GHK sequence. It has been investigated in connection with collagen-associated activity and dermal repair-related responses [33]. In some formulations, Pal-GHK is combined with palmitoyl tetrapeptide-7. Together, these peptides have been associated with ECM support and, in some reports, they have been linked to the modulation of inflammation-associated processes that may indirectly influence dermal aging [13,34,35].

Mechanistically, these peptides are used on the assumption that short synthetic sequences may reproduce part of the signaling activity of the larger paternal proteins [70,72]. By influencing fibroblast behavior, these peptides are proposed to shift the local balance toward matrix synthesis and maintenance [72]. However, the specific receptors involved and the downstream signaling pathways they activate are not always clearly defined [14,74]. In many cases, the mechanistic evidence is stronger at the level of observed cellular responses than at the molecular level and pathway elucidation [16,17]. Nevertheless, this group of peptides is central to many mechanistic explanations for anti-aging cosmetic formulations.

### 3.2. Modulation of Matrix Degradation and Repair-Associated Processes

The next important category involves the proposed mechanism in the modulation of ECM degradation while supporting tissue repair. In aging or stressed skin, structural integrity is affected not only by reduced synthesis of matrix components, but also by their increased breakdown [69,75]. This breakdown is attributed to the increased activity of matrix-degrading enzymes, particularly MMPs, as well as to inflammatory and stress-responsive signaling pathways [76,77]. Cosmetic peptides in this group are discussed for their proposed ability to reduce ECM breakdown, promote repair-related cellular activity, and help preserve dermal matrix structure [78].

In this context, MMPs are especially important [77,79]. These enzymes contribute to the degradation of collagen, elastin, and other ECM constituents. Their expression is often increased in aged or photoexposed skin [76]. Among them, MMP-1, MMP-3, and MMP-9 have received particular attention in skin aging because they serve as the key regulators of ECM remodeling. MMP-1 initiates fibrillar collagen cleavage, while MMP-3 and MMP-9 contribute to the subsequent degradation of collagen fragments and other matrix-associated proteins [77]. UV exposure and oxidative stress activate the mitogen-activated protein kinase pathways (MAPK), facilitating the upregulation of MMPs [80]. Many mechanistic discussions surrounding cosmetic peptides that influence the ECM focus on whether these molecules can reduce MMP expression or activity, either directly or indirectly, thereby slowing ECM degradation [81,82].

This concept is also closely linked to repair-associated cellular responses. Skin homeostasis depends on balanced matrix turnover, with matrix breakdown matched by renewal. Disruption of this balance can gradually contribute to visible signs of skin aging, including reduced firmness and wrinkle formation [6,69,76]. Tripeptide-10 citrulline (Decorinyl^®^) is a good example of a peptide with proposed functions in the matrix repair domain. Decorin is a small leucine-rich proteoglycan in the ECM. It interacts with type I collagen and helps regulate collagen assembly, spacing and size. Tripeptide-10 citrulline is expected to facilitate the activities associated with collagen and decorin, improving collagen fibrillogenesis. Nevertheless, the affinity and binding site of this peptide for its target, and whether it competes with endogenous decorin, are still under investigation [36,37,83].

In some cases, these effects are discussed alongside the modulation of inflammatory mediators or stress-responsive pathways that indirectly influence ECM turnover. For example, ultraviolet exposure and other environmental stressors can stimulate signaling cascades that increase the production of pro-inflammatory cytokines and matrix-degrading enzymes [77,79]. Under such conditions, a peptide that is thought to attenuate the upstream signals could, in principle, help reduce downstream matrix damage even if it does not act directly on structural proteins themselves. This distinction is important, since cosmetic peptides are often thought of as having matrix-preserving effects through multiple overlapping routes. These may include suppression of degradation, support of repair-associated processes, and indirect regulation of the signaling networks that govern these outcomes [81,82,84].

Overall, peptides discussed within this mechanistic category are thought to support skin structure by limiting ECM degradation. They may also influence broader repair-associated processes that help maintain dermal integrity over time.

### 3.3. Neuromodulatory Mechanisms Related to Expression Lines

Another mechanistic category involves peptides proposed to act through neuromodulatory processes that are associated with expression lines. Unlike peptides associated with ECM support or repair-associated pathways, neuromodulator peptides can influence the appearance of lines that form through repeated facial movement [16,63]. In cosmetic contexts, these peptides are often used as topical ingredients intended to soften the visible effects of muscle contraction-associated skin folding, particularly in areas such as the forehead and periocular region. Dynamic skin wrinkles commonly develop in highly mobile areas. Repetitive contractions cause recurring folding of the overlying skin. Moreover, these contractions contribute to the conversion of temporary expression lines into more persistent wrinkles [64].

In brief, neuromuscular transmission begins when neuronal stimulation opens voltage-gated calcium channels at the presynaptic nerve terminal. Calcium entry promotes assembly of the soluble N-ethylmaleimide-sensitive factor attachment protein receptor (SNARE) complex. The SNARE complex consists of several essential proteins, including synaptosomal-associated protein of 25 kDa (SNAP-25), syntaxin, and synaptobrevin/vesicle-associated membrane protein (VAMP), which mediate the docking and fusion of acetylcholine-containing synaptic vesicles with the presynaptic plasma membrane [85]. The subsequent release of acetylcholine leads to membrane depolarization and muscle contraction by interacting with nicotinic acetylcholine receptors on muscle cells [16,86,87].

Neuromodulator peptides are proposed to target different stages of neuromuscular transmission and muscle contraction. Depending on the peptide, the proposed mechanisms include inhibition of SNARE complex formation, reduction of presynaptic acetylcholine release, attenuation of calcium-dependent signaling involved in neurotransmitter exocytosis, and modulation of nicotinic acetylcholine receptor activity at the neuromuscular junction [16,38,88]. These peptides are frequently compared, sometimes too broadly, with botulinum toxin-like approaches, where the SNARE proteins are cleaved by foreign molecules to prohibit the release of acetylcholine. However, such comparisons should be made cautiously. The proposed effects of neuromodulator peptides are far more subtle and heavily depend on the conditions used. Also, their molecular mechanisms are much less understood [29].

One of the best-known examples of neuromodulators is acetyl hexapeptide-8 mentioned above. It is a synthetic peptide frequently included in cosmetic formulations and has been proposed to reduce the appearance of expression lines by temporarily interfering with the formation of SNARE to reduce the calcium-dependent neurotransmitter release, hence slowing down skin movement [16,63,85,89,90]. Other examples include Waglerin-1 and the dipeptide diaminobutyroyl benzylamide diacetate (SYN^®^-AKE). These peptides are proposed to modulate nicotinic acetylcholine receptor activity, thereby reducing neuromuscular transmission and muscle contraction that help diminish the appearance of expression wrinkles [38,39].

Mechanistically, these peptides are discussed as potential modulators of signaling events involved in neuromuscular communication, rather than as agents that act primarily through ECM support. The underlying rationale is that even a modest reduction in contraction-related signaling at or near the skin surface could lessen the repeated micro-movements that contribute to the formation of expression lines over time [16]. However, this interpretation requires caution [16]. For a topically applied cosmetic peptide to produce a neuromodulatory effect, it must penetrate the skin, remain sufficiently stable, reach the relevant site of action, and achieve a high enough local concentration capable of affecting the target pathway. These requirements are not trivial [17,29]. Importantly, direct experimental evidence that topically applied acetyl hexapeptide-8 reaches the relevant neuronal or neuromuscular compartment is currently lacking, and available penetration studies have reported little penetration beyond the superficial skin layers [16,29]. Available evidence suggests that skin delivery remains one of the main limitations for this class of ingredients. As a result, neuromodulatory cosmetic peptides should be described as having proposed, formulation-dependent effects rather than as direct topical equivalents of injectable neuromodulators [30,91].

### 3.4. Modulation of Inflammation, Oxidative Stress, and Related Signaling

Chronic inflammation and oxidative stress can disrupt skin homeostasis and accelerate processes associated with skin aging. Several cosmetic peptides have been investigated and proposed for their ability to influence these processes through the modulation of inflammation, antioxidant defenses, and related cellular signaling pathways [92,93]. In skin biology, these processes are closely connected to visible aging, especially when the skin is repeatedly exposed to UV radiation, pollution, or other environmental stressors [93]. Persistent activation of inflammatory mediators and oxidative pathways can alter fibroblast and keratinocyte behavior, disturb tissue homeostasis, and contribute to matrix degradation, in addition to reduced elasticity, wrinkle formation, and uneven skin appearance [94]. In this context, some cosmetic peptides are used as ingredients that may help limit pro-inflammatory signaling, reduce oxidative injury, or support cellular responses associated with tissue protection [95,96]. These effects should still be presented cautiously, since much of the evidence comes from in vitro or ex vivo studies only [96].

For example, environmental stress, especially UV exposure, increases the formation of reactive oxygen species (ROS), which subsequently activate MAPKs, allowing these kinases to remain active. Overactive MAPKs then trigger the activator protein 1 (AP-1) transcription factor, which is involved in the transcription of MMP genes, leading to ECM degradation [97,98]. ROS also activate nuclear factor kappa B (NF-κB), a master regulator of over 100 gene products, including pro-inflammatory cytokines such as tumor necrosis factor-α (TNF-α), interleukin-1 (IL-1), interleukin-6 (IL-6), and interleukin-8 (IL-8), which contribute to immune and inflammatory responses. Activation of the NF-κB complex promotes its translocation into the nucleus, leading to the overexpression of pro-inflammatory cytokines. This promotes the expression of MMPs, which are responsible for ECM degradation [99,100]. Nuclear factor E2-related factor 2 (Nrf 2) is another translocatable regulator that is expressed under different stress conditions. The Nrf 2 and Kelch-like ECH-associated protein 1 (Keap 1) pathway protects cells from oxidative stress caused by ROS [101]. In healthy cells, Keap1 interacts with Nrf2, leading to its rapid ubiquitination. Therefore, the expression of antioxidant genes is constitutively suppressed by the degradation of Nrf2. It has been demonstrated that disruption of the Keap1–Nrf2 interaction leads to the activation and stabilization of Nrf2 [102]. As a result, the expression of antioxidant proteins regulated by Nrf2 reduces ROS levels, thereby potentially reducing the activity of inflammatory cytokines.

Inflammation and oxidative stress are often mechanistically interconnected. Environmental stress can increase the production of ROS, activating stress signaling pathways such as MAPK mentioned above, resulting in the overexpression of cytokines and MMPs [92,98]. This, in turn, can further amplify oxidative imbalance and therefore impair fibroblast activity, and reduce the integrity of the ECM [75]. As a result, a self-reinforcing cycle of oxidative damage, inflammation, and tissue degradation can be established. In this context, a peptide capable of modulating one part of this network could, in principle, exert broader downstream effects on skin appearance and tissue maintenance. For this reason, peptides in this category are sometimes framed not only as anti-inflammatory or antioxidant agents, but also as modulators of the wider signaling environment that shapes cutaneous aging-related processes.

It has been shown that multiple peptides have the ability to reduce inflammation by inhibiting the enzymes that participate in the MAPK/AP-1 pathway [28,103,104,105]. In contrast, other peptides including seahorse-derived antioxidant peptide 2 (SHP2), cell-permeable marine peptides WP5, LW5 and YY6, and GHK-Cu, work in the opposite way to those involved in the NF-κB pathway. They are thought to disrupt the function of the Nrf2-Keap1 complex, resulting in the overactivation of Nrf2, which subsequently moves into the nucleus, promoting the overexpression of genes such as heme oxygenase-1 (HO-1), which is involved in antioxidant defenses. In this way, they may reduce ROS-dependent NF-κB signaling to minimize the activity of inflammatory cytokines [40,41,106,107].

In skin exposed to intrinsic aging or environmental stress, increased activity of these pathways can contribute to tissue damage and enhanced expression of matrix-degrading enzymes [76,108]. Cosmetic peptides proposed to act on these systems are therefore thought to attenuate pro-inflammatory or oxidative signaling, thereby supporting a more favorable cellular environment for skin maintenance [59,109]. However, as with other cosmetic peptides, the exact mechanisms involved and the extent to which topically applied peptides can meaningfully modulate these targets remain uncertain and require further investigation.

### 3.5. Effects on Pigmentation and Other Specialized Pathways

A final proposed mechanistic group consists of peptides that modulate skin pigmentation pathways. Skin tone is shaped by coordinated interactions between melanocytes and keratinocytes, enzymatic pathways involved in melanogenesis, and extracellular signals that regulate melanin production and distribution [110,111]. In brief, under UV exposure or certain other stimuli, paracrine mediators such as α-melanocyte-stimulating hormone (α-MSH) are released from keratinocytes and interact with receptors on melanocytes. Then, adenylyl cyclase, protein kinase A, intracellular cAMP, and its response element-binding protein (CREB) are activated to stimulate downstream signaling cascades, including the expression of microphthalmia-associated transcription factor (MITF). This transcription factor targets proteins including tyrosinase, which plays a critical role in melanosome formation, maturation, and transport, as well as in the regulation of melanin production [112,113,114,115,116]. Uneven pigmentation is a common cosmetic concern. Consequently, peptides targeting these pathways are of particular interest for brightening and tone-evening applications [66]. Unlike mechanisms centered mainly on dermal structure, peptides in this category are usually considered in terms of their effects on pigment-associated cellular communication, melanogenic enzyme activity, or melanosome handling. Depending on the peptide and its proposed target, these effects may involve modulation of melanocyte signaling pathways, regulation of enzymes such as tyrosinase, or interference with the transfer and processing of melanin-containing melanosomes in keratinocytes [117,118]. In cosmetic formulations, these actions are typically presented as relatively selective ways of influencing pathways that contribute to the visible distribution or intensity of pigmentation, although the strength of evidence varies considerably between individual peptides [117,119].

RMNE1 and its derivative, DualPep-Shine, have been reported to reduce melanin synthesis by inhibiting MITF function [42], whereas pentasodium tetracarboxymethyl palmitoyl dipeptide-12 (PTPD-12) aims to increase melanosome degradation, contributing to a reduction in melanin [43]. Additionally, the peptides oligopeptide-68 and AOP-P1 have been studied for their potential roles in regulating pigmentation-related signaling upstream of melanin production [120,121,122].

Peptides may also influence other specialized pathways that do not readily fit into the mechanistic groups outlined above. These may include, among others, effects related to barrier-associated signaling, antimicrobials, or pathways linked to more narrowly defined cosmetic outcomes [123,124,125]. The activities of such peptides are not discussed in the current study.

Overall, the mechanistic evidence for cosmetic peptides is still quite uneven. For some peptides, biological effects have been observed, but the direct molecular target, receptor, or downstream signaling pathway is still not well defined. In other cases, a possible mechanism has been suggested, but it is not clear whether a topically applied peptide can reach the required site in the skin at a high enough concentration. Therefore, observing a cellular or cosmetic effect does not necessarily mean that the proposed molecular mechanism has been confirmed. More work is still needed to directly validate targets and pathways, examine skin penetration, and confirm peptide activity under conditions that more closely reflect intact human skin.

## 4. Limitations of Current Peptides

### 4.1. Limited Mechanistic Insights

Despite their growing use in cosmetic formulations, current peptides still face several limitations that affect how their activity should be interpreted. One important issue is that the mechanistic evidence remains incompletely understood [10,16]. In some studies, peptides are associated with visible outcomes such as improved hydration, elasticity, or reduced wrinkle appearance. However, the molecular pathways responsible for these changes are not always clearly defined [10,29]. This makes it difficult to determine whether a given effect is driven by a specific receptor-mediated or intracellular pathway, by broader changes in skin physiology, or by the formulation as a whole [30]. The distinction matters. A peptide may show a desired activity in vitro, but its performance in a finished topical product can depend on stability, concentration, and interactions with other ingredients. As a result, the observed cosmetic benefit may not always be attributable to the peptide alone [126,127,128]. Limited mechanistic clarity also makes rational improvement more difficult. When the relevant target is known, such as a specific receptor, enzyme, or signaling complex, peptide design can be refined to improve binding, stability, localization, or delivery to the relevant site of action [25,129]. In contrast, when the mechanism remains poorly defined, optimization is often more empirical, making it harder to predict which sequence modifications or formulation changes will improve activity.

### 4.2. Meaningful Delivery Systems

Another important limitation is peptide delivery. For a cosmetic peptide to have a meaningful biological effect, it must reach the designed cellular location of action in sufficient concentration. Meeting this requirement is not an easy task [29,30]. The stratum corneum is a major barrier, and many peptides, particularly those that are larger, highly hydrophilic, or susceptible to proteolytic degradation, may experience limited penetration under typical topical conditions. Results obtained in cell culture or other controlled experimental settings are often produced under conditions that do not truly reflect the biology of intact human skin [17]. In topical use, peptide delivery can be influenced by factors such as the formulation vehicle, peptide concentration, skin condition, frequency of application, and the presence of competing ingredients [14]. Consequently, a peptide that appears active under controlled laboratory conditions may show weaker or less reproducible effects when incorporated into a finished cosmetic product [91].

### 4.3. Stability

Peptide stability is another important limitation. Many peptides are chemically and enzymatically vulnerable, and their activity can be affected by various environmental factors including oxidation, hydrolysis, temperature and pH [130,131]. They may also interact with other formulation components in ways that alter their structure, availability, or activity. This creates a practical challenge for cosmetic development [30]. A peptide may perform well in vitro, but keeping that same activity in a finished cosmetic product over time is not always easy [25,29]. This can complicate product development and may help explain why efficacy differs from one formulation to another.

### 4.4. Clinical Evidence

The clinical evidence for many cosmetic peptides also remains limited. Although some peptides have been evaluated in small human studies or formulation-based assessments [126], solid human trial data are still missing for many of them. Large, well-controlled, and independently replicated clinical studies are uncommon [10,132]. The studies that do exist often suffer from small numbers of participants, short follow-up periods, or limited methodological detail. In some cases, the evidence is also linked to proprietary products, making it harder to judge the specific contribution of the peptide itself [10]. When a complete formulation containing multiple active ingredients is evaluated, observed clinical effects cannot necessarily be attributed to the peptides themselves. Unless an appropriate comparator formulation differing only in the presence of the peptide is included, the effects of other ingredients, the vehicle, or interactions between formulation components cannot be excluded. Differences in peptide sequence, concentration, formulation design, treatment duration, skin type, and study endpoints further complicate direct comparisons across studies. As a result, it is difficult to draw a definite conclusion about the clinical efficacy of many cosmetic peptides [126,127,128].

Notably, clinical findings for cosmetic peptides have not always been positive. For example, in a double-blind study of acetyl hexapeptide-8, no significant differences in wrinkle scores or skin-age measurements were found between the side treated with an acetyl hexapeptide-8-containing serum and the side treated with the corresponding serum without acetyl hexapeptide-8 after four weeks [133]. Similarly, in a randomized study of topical GHK-Cu following CO_2_ laser resurfacing, computer analysis and blinded evaluators did not find significant improvements in wrinkles or overall skin quality compared with the control treatment. Patient satisfaction, however, was higher in the GHK-Cu group [134]. These studies do not mean that these peptides are ineffective under all conditions, but rather they may show that positive mechanistic or preclinical findings do not always result in clear clinical improvements.

The limited number of controlled studies is also seen in recent systematic analyses. A 2026 systematic review and meta-analysis identified 19 randomized controlled trials evaluating oral or topical peptides for skin aging, but only two of these studies evaluated topical peptide formulations. The overall effect on wrinkle reduction was mainly driven by oral peptide studies, while the topical subgroup showed a smaller and non-significant effect [135]. This further shows the need for more well-controlled studies specifically examining topical cosmetic peptides.

### 4.5. Target Accessibility

It should also be noted that not all targets are equally reachable and practical in the cosmetic setting [29,30]. Some proposed targets may be difficult to access through topical delivery, especially when meaningful activity would require penetration into deeper skin layers [91]. Intracellular entry and sustained exposure at sufficient concentrations might be needed for certain targets [136,137]. Other targets may be biologically relevant but too narrow to produce a meaningful visible effect [138]. In some cases, a pathway may influence one part of skin physiology, but its contribution to the final cosmetic outcome may be modest when considered within the broader network of aging-related changes [139]. The effect may also be highly context-dependent, varying with cell type, skin condition, age, environmental exposure, and the presence of other signaling inputs [92,94]. The practical reachability of these targets should therefore be supported by sufficient validation before reliable cosmetic benefit is assumed [17].

### 4.6. Regulatory and Commercialization Considerations

Regulatory requirements are also important when a new peptide is developed for cosmetic use. These requirements differ between countries and can depend on the claims made for the final product. In the United States, products making claims related to the structure or function of the skin may be regulated as drugs rather than only as cosmetics [140]. Regulatory frameworks for cosmetics also differ internationally, including in Canada [141].

There are also practical issues involved in commercialization. A peptide that shows good biological activity still needs to be produced at a reasonable scale and cost. It also needs sufficient stability, formulation compatibility, and safety for use in a final product. These issues have been identified as important considerations in the development of peptide-based cosmeceuticals. Therefore, successful peptide discovery alone does not necessarily mean that the peptide will be practical for use as a cosmetic ingredient.

Together, these limitations point to the need for better peptide design, improved delivery approaches, stronger mechanistic studies, more in-depth clinical validation and greater consideration for regulatory and commercialization requirements.

## 5. Classical Strategies for Discovering New Peptides

Before the widespread adoption of AI and advanced machine-learning methods, new bioactive peptide discovery relied on a variety of classical approaches, including experimental isolation, screening-based methods, and rational or computational design. These approaches laid much of the groundwork for the development of currently available functional peptides. They also continue to play an important role in current peptide discovery efforts. The methods discussed in this section are not meant to cover all strategies that have been used in the field. Instead, they highlight a representative set of classical approaches that have commonly been used to identify new functional peptides. Although the boundaries between these approaches are not always strict, general peptide-discovery methods can be organized into three broad categories: natural discovery and its derivatives, library-based screening, and rational computational design.

### 5.1. Experimental Discovery from Natural and Biologically Derived Sources

One of the most established methods for peptide discovery involves the identification of active sequences from natural and biologically derived sources [142,143]. In contrast to de novo design, this approach begins with peptides or precursor proteins that already exist within biological systems. Candidate sequences may be isolated from natural peptides, generated through the proteolytic processing of larger proteins, or identified from a protein complex. Since they interact with their corresponding partners in vivo, they may already exhibit the structural or physicochemical stability that supports the interactions with the target of interest, including receptor binding, enzyme modulation, membrane association, as well as participation in ECM formation.

Natural sources have long served as an important reservoir for peptide discovery. Bioactive peptides have been identified from a wide range of organisms and materials, including animals, plants, microbes, marine organisms, and human tissues [144,145,146,147]. In some cases, the peptide of interest is isolated directly as a naturally occurring molecule, whereas in others it is obtained as a fragment released from a larger precursor protein through enzymatic digestion, hydrolysis, or related processing steps. These peptides may be of interest because they already exhibit activities relevant to cosmetic applications, such as antimicrobial effects, antioxidant properties, support of barrier function, or modulation of cellular responses linked to skin appearance and condition. One example is the human cathelicidin peptide LL-37, an endogenous host-defense peptide expressed in human skin [148]. It has attracted attention due to its antimicrobial and immunomodulatory properties and its skin repair responses [149,150]. Although it is not primarily used as a traditional cosmetic peptide, it illustrates how naturally occurring peptides can reveal biologically relevant functions and provide templates for the development of new peptides.

Another well-known example, which has been discussed above, is GHK found in human plasma, saliva, and urine. It has also been associated with the proteolytic processing of ECM proteins, including secreted protein acidic and rich in cysteine (SPARC) [59,151]. SPARC-derived copper-binding peptides containing the GHK-related motif have been shown to stimulate angiogenesis, supporting the idea that matrix-derived GHK-containing peptides can participate in tissue-remodeling responses after proteolytic breakdown [151]. As discussed above, GHK-Cu has also attracted interest because of its reported associations with tissue remodeling and skin-related applications [59].

A related but more targeted strategy has been to explicitly derive peptides from biologically relevant protein domains and motifs [152,153]. Unlike the approach described above, which begins with naturally occurring peptides or peptide-containing mixtures that display a desirable activity, this strategy starts with a larger parental protein that is already known to participate in a targeted activity, such as structural support, signaling, repair or other skin-related functions [154]. Short sequences are then selected from functional regions of the parent protein with the expectation that they may retain part of its biological activity. This approach has been particularly influential in cosmetic peptide development, where motifs derived from ECM proteins, signaling molecules, or other endogenous sequences have often served as starting points for active ingredient discovery [71,152]. One well-known example is KTTKS, a pentapeptide derived from the C-terminus of type I procollagen that has been widely considered as a starting template for peptide development. The peptide was reported to stimulate ECM production in fibroblasts and was later chemically modified through palmitoylation to achieve better delivery [71,74]. This example illustrates how a natural peptide can first be identified from a relevant parent protein and then further optimized for cosmetic applications.

Peptides have also been identified more empirically from complex biological extracts and mixtures [155,156,157]. In this type of approach, peptide-containing extracts are first screened for a desired biological activity and then separated into smaller fractions. Fractions that retain activity are examined further, allowing the active component or components to be isolated and structurally characterized [158,159,160]. One advantage of bioassay-guided fractionation is that it does not require a fully defined molecular target at the outset. Instead, discovery is guided by the observed biological response. This can be useful when the relevant mechanism is uncertain, since it may reveal active peptide candidates that would not have been predicted from existing mechanistic knowledge. A drawback, however, is that the initial activity is often observed in a mixture rather than in a purified sample. As a result, additional work, sometimes very tedious, is needed to determine which peptide is responsible for the observed activity and whether the peptide itself is truly driving the intended effect. An example of this strategy is the identification of Tyr-Gly-Asp-Glu-Tyr (YGDEY) from tilapia skin gelatin hydrolysates, where peptide-containing hydrolysates were screened and the active peptide was subsequently isolated and characterized for anti-photoaging-related activity [44].

Overall, natural and biologically derived sources remain an important starting point for peptide discovery because they provide sequences that are already associated with biological functions or interactions. These sequences can therefore offer useful templates for identifying new activities and developing peptide cosmetic ingredients. However, discovery from natural sources can also be time-consuming and resource-intensive, particularly when working with complex mixtures. Extensive purification, structural characterization, and functional testing may be required to determine which peptide is responsible for the observed activity. In many cases, further modification is also needed to improve properties such as stability, specificity, or delivery before the peptide can be considered for practical applications.

### 5.2. Library-Based Screening and Display Technologies

Another important strategy for peptide discovery is the use of library-based screening and display technologies in the wet lab. In these approaches, large collections of diverse peptide sequences are generated and then screened for binding (or functional activity) against a target protein of interest [161,162,163]. Unlike starting from a known endogenous peptide or derived protein fragments, library-based methods enable the identification of candidate peptides from a random sequence space. This can reveal peptides that would not have been predicted from existing biological knowledge alone. This makes library-based approaches especially useful for discovering peptides with high affinity when a defined target is available. In short, these methods search a pool of random sequences to identify those with specific properties.

Several platforms have been used for this purpose, including phage display, yeast display, ribosome display, and synthetic peptide libraries [163,164,165,166]. Although these methods differ in how peptide diversity is created and linked to screening, they follow the same basic principle. First, a large set of random peptide sequences (peptide library) is generated. Each peptide is physically associated with a system that carries or encodes it. This allows individual peptides with desirable properties to be traced back and identified after screening. The library is then exposed to a target protein of interest. The peptides that show the desired binding are retained. The retained candidates are then recovered, amplified, and subjected to additional rounds of selection to further enrich the pool for the top binding peptides. A major strength of these approaches is their ability to sample a broad and diverse sequence space in a systematic way. Another advantage is that they can uncover active sequences that would have been difficult to predict from existing biological knowledge alone. For this reason, they are particularly useful for identifying lead peptides for a well-defined target [167]. Their main limitation is that activity observed during screening does not always reflect activity in a biological setting. Additional validation and optimization are needed before such peptides can be considered strong candidates [168,169]. Nevertheless, these methods offer an attractive starting point for identifying lead peptides that can be further characterized and improved.

In cosmetic peptide discovery, library-based approaches have been useful not only for finding target binders, but also for identifying sequences with more specific biological functions [45,170,171]. Rather than simply generating a large number of candidates, these methods can also help identify peptides that can modulate the activity of certain pathways and cellular responses. For example, phage display was used to identify the peptide P12-3A, an inhibitor of insulin-degrading enzyme. It was shown that P12-3A enhances insulin-induced collagen-related responses in skin fibroblasts and in doing so can contribute to wound-healing and skin health [45]. Likewise, phage display libraries were used to identify P15-1, a peptide that mimics the receptor for hyalu-ronan-mediated motility (RHAMM) and regulates hyaluronan-related signaling [46]. This peptide is thought to modulate wound repair responses after topical application.

Overall, library-based screening provides a useful way to discover peptides without having to begin from a known natural sequence or biological motif. It can identify peptides with strong binding or functional activity from a very large sequence space and can therefore provide useful starting points for further development. However, the conditions used during screening are still simplified compared with a biological system. Peptides identified in this way need additional testing to determine whether their binding, specificity, stability, and biological effects are maintained in cells, tissues, and eventually in vivo.

### 5.3. Rational Design and Classical Computational Approaches

Rational design and classical computational approaches aim to identify useful peptides by drawing on existing biological, biochemical, or structural knowledge rather than relying only on large-scale experimental screening. A central idea in this area is mimicry [172,173,174]. In this context, a peptide is designed to reproduce a functional feature of a natural molecule, such as a receptor-binding motif, a matrix-derived signaling fragment, or a protein region involved in a protein–protein interaction (PPI) [175]. The reasoning is that if a defined part of a natural protein or peptide is responsible for a particular activity, that feature may be captured in a shorter synthetic sequence and adapted for practical use. These sequences are then logically modified for improved activity. Several related strategies fall within this general framework [176]. In some cases, short peptides are derived directly from endogenous proteins or natural ligands that already have a known biological role. In other cases, a larger active region is narrowed down to a smaller motif that still retains the desired function. A known binding sequence may also be grafted onto a simpler scaffold, or an existing bioactive peptide may be used as a lead and then modified further. These modifications can include residue substitutions, sequence truncation, terminal changes, cyclization, or lipidation [177,178]. Depending on the objective, such changes may be introduced to improve stability, preserve a favorable conformation, increase resistance to enzymatic degradation, enhance solubility, strengthen binding, or improve delivery.

This distinguishes rational design from random library screening. Library approaches explore large numbers of random peptides, whereas rational design begins with a candidate linked to its potential mechanism through previous studies [129,175]. That information may come from knowledge of ligand–receptor recognition, structural studies of protein interfaces, sequence conservation, or previous functional work on related peptides or proteins [179,180,181]. For this reason, rational design is usually more focused than empirical discovery. Rather than screening very large numbers of unrelated sequences, it begins with a candidate that is already linked, at least in principle, to the activity one hopes to obtain.

A good example is the design of Pal-KTTKS. The parent sequence KTTKS is considered a collagen-derived motif, and the peptide was developed as a synthetic topical agent intended to stimulate collagen-related responses [71]. The addition of a palmitoyl group produced a derivative with improved dermal stability and skin permeation [25]. This reflects the general workflow of rational peptide modification. A biologically meaningful motif is identified first. The sequence elements believed to underlie activity are retained, and the peptide is then modified to improve practical properties such as stability, affinity, or delivery. Similar logic is used more broadly when lead peptides are optimized by systematic residue replacement, alanine scanning, or the introduction of chemical groups that improve conformation or resistance to degradation [177,182,183].

Computational methods have made rational design more systematic and more efficient [184,185]. These conventional in silico approaches are computational design tools and are not, by themselves, considered AI-based methods (see below). In peptide work relevant to cosmetics, they have been used to identify candidate motifs, predict physicochemical properties, model peptide–target interactions, and rank variants before synthesis [152]. This is especially useful when the biological target is already known. In such cases, molecular docking and related in silico methods can help compare candidate sequences, estimate how they may interact with the target, and reduce the number of peptides that need to be tested experimentally [186]. An example comes from the α-conotoxin field, a family of neurotoxic peptides from cone snail venom. While it is distinct from botulinum neurotoxin in both structure and mechanism, α-conotoxins also act on cholinergic pathways [187]. In one study, a co-crystal structure of α-conotoxin LsIA bound to acetylcholine-binding protein was used to guide the design of peptide analogs and define the structural features required for α3β4 nicotinic acetylcholine receptor antagonism [188].

Overall, rational design and computational approaches provide a more focused way to discover and optimize peptides by using existing biological and structural information. They can reduce the number of candidates that need to be tested experimentally and help guide modifications before synthesis. However, their success depends strongly on how well the target, binding site, and relevant molecular interactions are understood. Computational predictions are also only as reliable as the models and input data used, so experimental testing is still needed to confirm binding, activity, and biological effects.

## 6. AI-Based Strategies for Peptide Discovery

AI-guided discovery represents a recent direction in peptide research. It offers an important step forward in peptide discovery and design. In this review, we use the term AI-based methods to refer to approaches that employ machine learning, deep learning, generative models, or related data-driven algorithms to learn patterns from existing data and use them for prediction or peptide design. Conventional computational methods such as molecular docking, molecular dynamics, molecular mechanics, and traditional scoring functions are not considered AI on their own, although they may be combined with AI methods within integrated design workflows. Rather than relying on known functional motifs, direct structural reasoning, or large-scale screening experiments, these approaches use machine learning and related methods to identify patterns in existing peptide and/or PPI data [24,189,190]. These patterns can then be used to predict useful properties and, in some cases, to generate new candidate sequences. Depending on the design goal, the predicted properties may include biological activity, binding potential, toxicity, stability, solubility, or other features relevant to peptide performance [191,192]. In this way, AI-based methods can not only support the selection of promising candidates from existing datasets but also propose novel sequences for further study.

One advantage of AI-guided methods is their ability to explore a much broader peptide sequence space than would normally be practical using conventional approaches alone. By investigating candidates before synthesis, they can reduce the number of peptides that need to be experimentally tested. This makes early-stage discovery more time- and cost-effective. They may also uncover candidates that would not have been readily identified through traditional rational or screening-based approaches alone [189,190,193]. For these reasons, AI-guided peptide design has received considerable attention in the medical field, particularly in diagnostics and therapeutics, where the need for efficient identification of functional and selective peptide candidates is particularly important [194,195,196]. By contrast, its direct application to cosmetic peptide discovery is just beginning to be discussed. To our knowledge, the direct application of AI-guided peptide discovery to cosmetic research remains limited and is best viewed as an emerging future direction, although cosmetic-focused studies and reviews suggest growing interest in applying data-driven peptide design to skincare, including anti-aging and skin-repair applications [197,198].

AI-guided peptide discovery and design is still in its infancy. Despite growing interest, relatively few AI-derived peptide candidates have undergone extensive experimental validation. AI-predicted peptide candidates require substantial experimental follow-up. Their predicted activity must be confirmed in relevant biological assays, and additional properties such as stability, toxicity, selectivity, and performance in the appropriate biological context must also be evaluated [24,193,199]. Progress is also limited by the quality and quantity of available training data and by the difficulty of capturing the full structural and biological behavior of peptides in silico [193]. Another consideration is sequence redundancy between training and test datasets. Highly similar peptides appearing in both datasets can result in information leakage and overestimate model performance. Similarity-aware splitting, in which closely related sequences are clustered and kept within separate training and test sets, can provide a more reliable assessment of the model. Also, since peptides are flexible molecules whose activity can heavily depend on environmental context, computational predictions do not always translate directly into biological performance. Nevertheless, AI-guided discovery already offers clear advantages as a powerful and rapidly evolving approach that can expand accessible sequence space, improve candidate prioritization, and make early-stage peptide discovery more efficient. At present, it is best viewed not as a replacement for classical peptide discovery strategies, but as a very promising complementary approach, which is becoming increasingly important for peptide research and design.

Direct applications of AI to cosmetic peptide discovery remain limited. A very small number of studies have used AI approaches to identify or optimize peptides specifically for skin or cosmetic applications, including the AI-assisted discovery of the naturally occurring anti-aging peptide pep_RTE62G [47] and the design of the antimicrobial peptide IK-16-1 for cosmetic preservative applications [48]. However, many of the more advanced demonstrations of AI-guided peptide design and target-specific generation have been developed in the therapeutic field. Consequently, therapeutic examples are discussed below to illustrate design strategies that could be transferred to cosmetic peptide design in the future, rather than to suggest that these approaches are already established in cosmetic applications.

**Figure 1:**
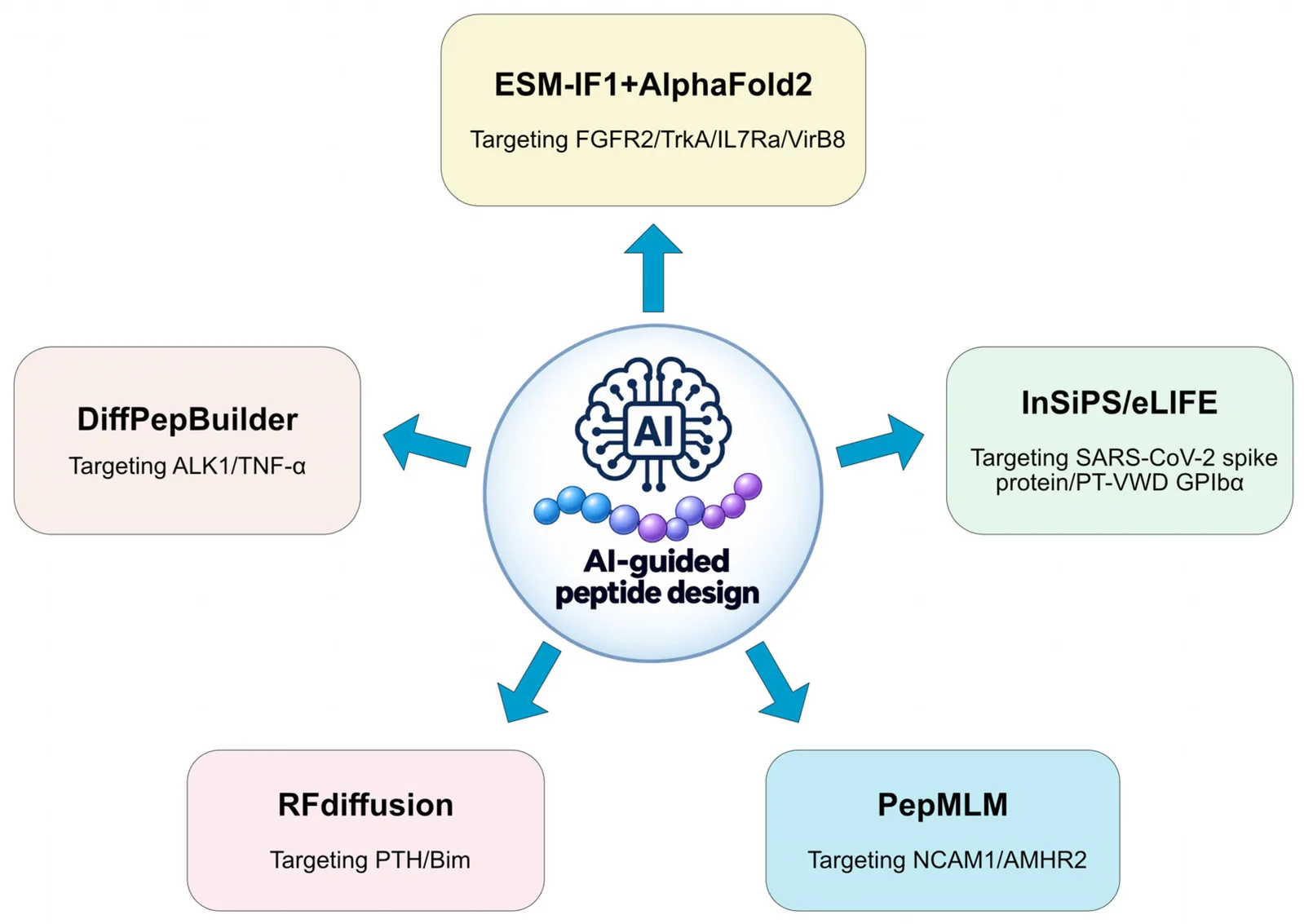
Selected case studies of AI-assisted peptide discovery and design discussed in the current study. Clockwise from the top: protein binder designed against FGFR2/TrkA/IL7Ra/VirB8 via ESM-IF1 along with AlphaFold 2 validation [200]; peptides designed against SARS-CoV-2 spike protein and GPIbα in PT-VWD by InSiPS/eLIFE [194,196]; peptide binder designs against NCAM1 and AMHR2 from PepMLM [201]; de novo design of binders against peptide hormones PTH and Bim via the RFdiffusion model [202]; de novo design of peptide binders against ALK1 and TNF-α using the DiffPepBuilder model [203].

Several case studies illustrating the practical application of computational approaches to peptide discovery and design are summarized in Figure 1. It includes examples with direct cosmetic applications as well as broader proof-of-concept studies demonstrating strategies that may be adapted for future cosmetic peptide development.

### 6.1. Structure-Based AI Approaches

In the classical view, PPIs are fundamentally formed by a 3D-based physical contact between two proteins [204,205]. Their formation depends on the geometric and chemical properties of the interacting partners, including conformation, charge distribution, hydrophilicity, hydrogen bonding, and the spatial arrangement of key side chains [206,207]. The strength and specificity of the interaction depend on how well these features match across the binding interface. Often, only a subset of residues makes the largest contribution to binding, but these residues act within a defined 3D context. Although only a subset of interface residues may contribute to most binding events, these residues function within a specific 3D environment that determines their orientation and accessibility [208,209]. The structural information, therefore, can play a central role in the design of peptides to mimic or disrupt PPIs.

Certain structure-based AI approaches use this 3D information to guide peptide discovery [175,200,203,210]. These methods begin with the structural and chemical features of a target protein. Their aim is to identify peptides that can bind a defined region on the protein surface, such as a pocket or other interface site likely to support binding. In some cases, the peptide is designed to fit into a local surface depression. In others, it is designed to reproduce the key contacts made by a natural binding partner [129,175]. In both settings, the design process is guided by the local architecture of the target surface and by the arrangement of residues that are most important for interaction. As a result, peptide candidates are selected or designed based on how well they match the geometry and chemical features of a specific site on the protein.

A typical workflow starts with a structural model of the target protein obtained from experimental methods, such as X-ray crystallography, nuclear magnetic resonance spectroscopy, or cryo-electron microscopy. A predicted model may also be used, but with limited reliability [211,212]. The next step is to identify a region of interest on the protein surface. This can be a known 3D binding site or a pocket predicted to contribute strongly to binding. Identifying such a site accurately is a key step in peptide discovery/design. This is not always a straightforward task, as many structural pockets remain insufficiently characterized [213,214]. AI-based methods can then be used to examine the geometry and chemistry of the site [203,215,216]. They will be utilized to guide peptide design that can fit into the pocket. This involves predicting favorable residue placements, estimating peptide binding poses, proposing new peptide sequences, and ranking candidates based on how well they match the structure. The overall goal is to generate peptides that are structurally and chemically compatible with the selected site.

These methods can be used in several ways. One approach starts with a short binding segment extracted from the interface that indirectly interacts with its nat-ural protein partner [175,217]. AI-guided optimization is then used to improve that segment by modifying residues expected to strengthen binding and stabilize the interaction. This can involve preserving the main contact pattern of the original segment while modifying other features such as side-chain composition and conformational adjustments. Another approach is de novo design, in which new peptide sequences are generated to complement the target surface without directly copying a natural binding segment [202,203,218]. In both cases, the design logic is the same. The peptide is shaped around the 3D features of the target site, with attention to steric fit and the placement of residues that can form favorable contacts. The main difference is whether the design begins from an existing biological interaction or from a newly generated sequence built to match the structural features of the target.

Recent studies have expanded this concept using several independently developed deep-learning approaches. For example, RFdiffusion uses a diffusion-based generative process to design new protein structures [219]. The model starts with a highly disordered or noisy representation and gradually generates a plausible 3D protein backbone while taking into account constraints such as the structure of a target protein or a desired binding site. Although RFdiffusion was developed primarily for protein rather than peptide design, related diffusion-based strategies have subsequently been adapted specifically for peptide design. DiffPepBuilder applies a similar idea more directly to peptide design using an SE(3)-equivariant diffusion model. Here, SE(3)-equivariance indicates that the model processes 3D structural information in a manner that is independent of how the peptide–target complex is formed or rotated in space [203]. The method can generate peptide structures and sequence together while considering their interaction with a defined protein target [203]. Other approaches have combined sequence-design methods with structure-prediction models. For example, Bryant and Elofsson integrated ESM-IF1 with AlphaFold2 to generate and evaluate peptide binders for defined protein interfaces [200]. Generative deep-learning methods have also been combined with more traditional structural refinement approaches, such as Rosetta FlexPepDock and molecular dynamics simulations. Such integrated workflows can refine and evaluate peptide–target interactions and have been used to generate experimentally validated target-specific peptide inhibitors [220]. Structure-based AI methods are especially useful when the target contains a well-defined pocket [221,222]. Such regions provide better geometric and chemical constraints for peptide design and can therefore make it easier to identify candidates with favorable structural complementarity. When a pocket or locally defined binding site is present, features such as conformation, depth, residue arrangement, charge distribution, and hydrophobic character can be used to guide peptide design in a more directed manner.

Despite these advantages, structure-based AI peptide design remains challenging [186,210,212,223]. Many protein surfaces involved in PPIs are relatively flat, flexible, and solvent exposed, which makes them harder to target than classical binding pockets. Peptide binding can also depend on conformational changes in the peptide, the target protein, or both. These dynamic features are difficult to predict with high confidence. The computational burden can also be substantial, especially when large numbers of candidate sequences, conformations, and binding poses must be evaluated.

Specificity is another important limitation. A peptide designed for one target site may still recognize similar surface features on other proteins, so off-target binding needs to be assessed carefully. This type of comparative analysis can be computationally demanding. Furthermore, the structure-based methods rely on the availability of reliable structural information, and whether a target contains a defined pocket or local binding site. Dependence on such local sites limits the range of protein regions that can be targeted. Even with these limitations, structure-based AI has made peptide discovery more targeted and more mechanistically grounded by allowing peptide design to be guided through the architecture of the target surface [224].

### 6.2. Sequence-Based AI Approaches

Sequence-based AI approaches build on earlier computational work showing that information relevant to protein binding can, in many cases, be extracted directly from the primary amino acid sequence [225,226,227]. In contrast to structure-based methods, these approaches do not require a resolved 3D structure of the target or the interacting interface. Unlike the traditional mode of PPIs, which typically depends on large interacting protein regions, resembling domain–domain interactions, sequence-based approaches rely on smaller sequence elements that mediate binding [228]. These interactions are therefore better described as a hook-and-loop type of interaction, in which a limited number of short regions drive protein association [229]. This provides an alternative framework for understanding interactions between proteins. The ability to work directly from sequence offers several practical advantages. Structural information for many proteins remains incomplete or unavailable, and even for a solved structure, the reliable relevant binding interface may not be available [225,230]. Avoiding this dependence on detailed structural models, sequence-based methods instead search for specific patterns in sequence that are associated with interactions. They utilize such patterns to predict interactions or, in more advanced cases, to help design new binders. In that sense, they represent a different route to peptide discovery, one that begins from sequence rather than structure. In contrast to structure-based approaches, these methods are faster and less computationally demanding. They are also more scalable, can achieve higher specificity through an inherent negative selection step, and can reach additional regions of the protein target, thereby expanding the druggable space [231].

An early and important step in this area was the notion that short sequence fragments that participate in PPIs may re-occur in different proteins, and these re-occurring patterns can therefore be used to infer new interactions directly from sequence [232]. This was significant as it suggested that one could extract interaction-relevant information without first solving protein structures or relying entirely on experimental screens. Follow-up work further developed and expanded this framework [233]. Later studies improved the method and applied it to large-scale prediction of PPIs, first in yeast [234] and then more broadly across other organisms [235], and eventually for the human interactome [236]. Other PPI prediction methods were also developed on the basis of sequence information [237,238,239,240]. Taken together, this body of work showed that sequence-based interaction could be applied at much larger scale than might have been expected.

The sequence-based mode of PPI has several practical advantages for peptide discovery [231,241,242]. On the one hand, it does not depend heavily on structural data, which makes it easier to apply across a wider range of targets. It is also faster and requires less computational power than structure-based approaches. Since it works directly with protein sequence, it is more scalable and better suited for screening large numbers of proteins or candidate binders in a relatively short time. This is especially useful when the goal is not only to study one interaction in detail, but also to search broadly for candidates or potential interaction sites across targets. Sequence-based approaches can also achieve higher specificity when an inherent negative selection step is built into the design process, allowing candidates with likely off-target interactions to be removed early. In addition, importantly, they can reach regions of a protein target that are not easily captured by pocket-focused structure-based methods, thereby expanding the druggable space within a given target protein. For these reasons, sequence-based approaches are particularly useful for peptide design and for large-scale screening across different targets [243,244].

Earlier studies were aimed mainly at predicting PPIs rather than designing peptides directly. Their importance for peptide design was therefore initially more conceptual than immediate. They showed that primary sequence alone can carry enough information to support meaningful predictions about binding [245,246]. That point is important, because once interaction-relevant features can be inferred from sequence, the same general logic can, in principle, be extended from prediction to design. This transition became clearer in later work, such as the development of InSiPS (later known as eLIFE) [247]. This tool moved beyond interaction prediction and used sequence-based PPI scoring together with an evolutionary optimization framework to generate synthetic binding proteins from random amino acid sequences. This study further demonstrated that sequence-based computational methods could be used not only to identify likely interactions, but also to actively search sequence space for new binding molecules with desired properties, including improved target preference and reduced off-target binding. Since then, other algorithms have also been developed that utilize the information from primary sequence [227,248]. Currently, the sequence-based framework is no longer limited to only asking whether two natural proteins may interact. It can now generate or prioritize candidate peptides with selective binding properties against biologically important targets. This shift from prediction to design has made sequence-based methods increasingly relevant to the development of diagnostics [196] and therapeutics [195]. This is especially important when high specificity is required and when large sequence spaces need to be searched rapidly and efficiently.

Another attractive feature of the sequence-based approaches is that they may extend beyond the de novo design of artificial peptides. The same logic for interaction detection can be used in reverse and as a query to search natural proteomes for related motifs or peptides with similar predicted binding properties [249]. For example, an artificial peptide designed by a sequence-based algorithm for severe acute respiratory syndrome coronavirus 2 (SARS-CoV-2) therapy was later used to discover a natural peptide with similar properties [195,249]. This is particularly interesting as it opens the possibility of discovering naturally occurring peptides with desirable properties after an artificial peptide is generated. For cosmetic applications, natural peptides may have additional appeal, as some consumers may perceive them as more natural and therefore less likely to cause side effects than artificially designed peptides. Natural peptides are reported to have lower immunogenicity, greater stability, and potentially faster regulatory approval [250,251,252], making them more attractive for cosmetic applications and regulatory approvals.

More recently, advanced protein language models have provided new routes to sequence-based peptide generation. For example, PepMLM fine-tunes the ESM-2 protein language model using data describing protein–peptide interactions [201]. The model takes the sequence of a target protein as input and uses the information learned from known interactions to generate new linear peptide sequences that may bind to that target. Unlike structure-based design methods, the generation step does not require a resolved 3D structure of the target. This illustrates how representations learned by large protein language models can be adapted directly to peptide design, providing an alternative to sequence-based approaches that rely on interaction-prediction scores.

Despite these advantages, sequence-based approaches also have inherent limitations [231,241,248]. Predictions based primarily on sequence can produce false-positive interactions, particularly when the sequence patterns associated with binding are not sufficiently specific. In addition, when used without complementary structural modeling, sequence-based methods generally provide limited information about the interface and structural basis of the predicted interaction. This makes it more difficult to determine which residues are directly involved in binding, how the peptide is oriented relative to the target, and whether the predicted interaction is structurally plausible. Their performance also depends strongly on the quality and diversity of the data used for model development. Models trained on limited or biased datasets may perform well on familiar sequence types but perform less reliably on targets that differ substantially from those represented in the initial training data. Furthermore, sequence-based predictions may not fully capture conformational changes or other context-dependent features that influence binding in biological systems.

Overall, sequence-based approaches offer a useful and practical route for cosmetic peptide discovery and design. They are especially attractive when structural information for skin-related targets is limited. Similarly, they are useful when large-scale screening is needed, or when one wants to search broadly across a vast sequence space in a relatively fast and efficient way. This is particularly relevant in cosmetic research, where there is interest in identifying peptides that may influence several processes linked to skin aging. At the same time, off-target binding and other unwanted effects need to be kept as low as possible. It should be noted that these approaches do not replace the need for experimental validation. They do not capture the exact contextual complexity of peptide behavior in skin. Still, their development from early interaction-prediction methods to later binder-design strategies clearly shows that useful design information can be extracted from sequence alone. That information can be used not only to generate artificial peptides quickly and efficiently, but also to identify naturally occurring candidates with desirable properties. In this sense, sequence-based AI provides a promising framework for expanding the repertoire of designed and natural peptides available for cosmetic applications.

Altogether, structure-based and sequence-based AI approaches have different strengths and limitations (summarized in Table 2). Structure-based methods can provide more direct information about the possible peptide–target interface, but they depend on having useful structural information. They are also computationally more demanding. Sequence-based methods can search much larger sequence spaces without requiring a resolved target structure, but they usually provide less information about the actual binding interface. In both cases, computational predictions are only an early step. Candidate peptides still need to be tested experimentally for binding and biological activity, followed by evaluation of selectivity, stability, toxicity, skin delivery, and performance in relevant formulations.

## 7. Future Directions in AI-Guided Cosmetic Peptide Discovery

**Figure 2:**
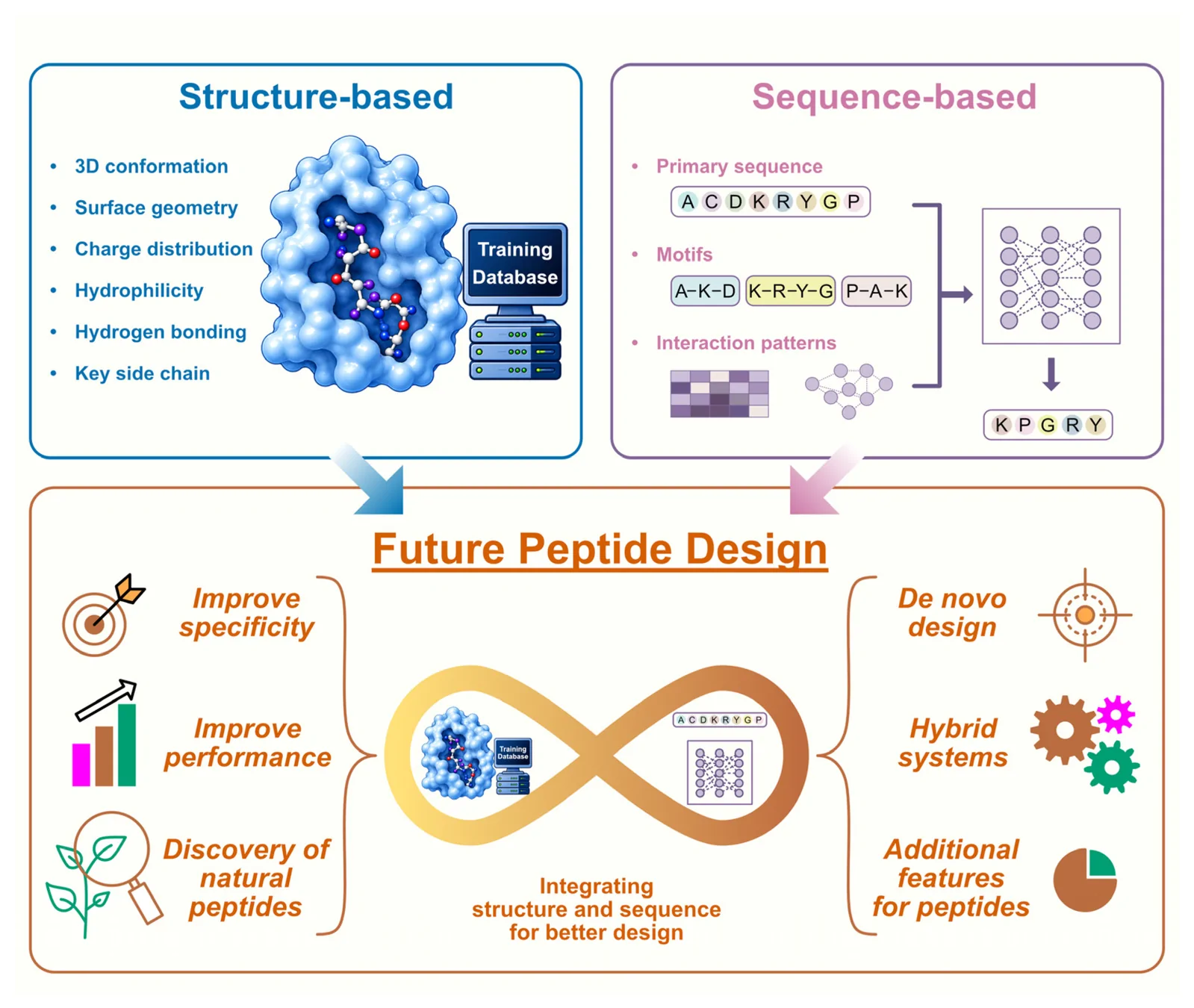
Potential contributions of AI to the future of cosmetic peptide design. Sequence-based methods can support broad peptide generation, screening, and optimization, while structure-based approaches can provide target-specific structural refinement. Combining these complementary approaches may facilitate the identification and optimization of selective, stable, and multifunctional cosmetic peptides.

This section considers possible future directions for the use of AI in cosmetic peptide discovery and design (Figure 2). Since the field is still at an early stage, these should not be viewed as firm predictions or a complete roadmap. Still, the strengths and current limitations of AI-guided methods already point to several likely directions for future work. These include improving peptides against known targets, developing candidates for newly identified targets, designing peptides with better selectivity and fewer undesired effects, mining natural peptides, and optimizing multiple peptide properties simultaneously. The aim here is not to predict exactly where the field will go, but to outline some realistic and scientifically grounded possibilities.

One likely direction is the use of AI to improve peptides directed against cosmetic targets that are already well known, for example, MMPs involved in collagen degradation [76]. Many such targets have been studied for years, and certain peptides aimed at some of them already exist. However, these peptides are not always optimized for selectivity and overall performance [10]. Therefore, a possibility is that AI could be used not only to identify the binders, but also to refine the existing peptide designs and generate improved variants. This may allow the development of peptides with stronger target preference, reduced off-target binding, and more favorable functional properties. Such an approach fits particularly well with sequence-based design, where rapid exploration of large numbers of variants and early elimination of less selective candidates are important advantages.

Greater consideration of specificity is likely to become a part of future design workflows. In cosmetic applications, activity alone is not sufficient if a peptide also interacts with undesired targets or pathways leading to undesirable effects such as irritation or other unintended biological responses [253,254]. For this reason, future AI-based design strategies may increasingly place selectivity at the center of the design workflow. Sequence-based approaches may be especially valuable, since they can incorporate negative selection steps and remove candidates with likely off-target interactions early in the design process [247]. For example, InSiPS (eLIFE) evaluates candidate sequences against both the intended target and a predefined set of non-target proteins, allowing candidates with predicted off-target interactions to be penalized or eliminated during optimization [247]. This strategy has subsequently been applied using human cell-surface proteins as a negative-selection set in the design of SARS-CoV-2-binding peptides and a selective peptide targeting disease-associated GPIbα [194,196]. Such approaches allow peptide design to optimize not only target recognition, but also non-recognition of potentially relevant off-target proteins.

AI may also become increasingly useful as new skin-related targets continue to emerge [255,256,257]. As research advances, additional proteins and pathways involved in skin aging, ECM regulation, pigmentation, inflammation, and barrier function will likely be identified [258,259]. For many of these targets, especially at an early stage, detailed structural information may be limited. Experimentally screening large numbers of peptides is very costly and slow. Under those conditions, AI-based methods could provide a meaningful starting point by generating and prioritizing candidate peptides more efficiently. This would make AI useful not only for improving existing peptide classes, but also for opening new directions in cosmetic peptide discovery as the biology of the skin becomes better understood.

Developing hybrid pipelines that combine sequence-based and structure-based AI methods is another promising direction [220]. These two approaches offer different strengths. Sequence-based methods are faster, easier to apply across large candidate spaces, and meaningful for broad exploration. Structure-based methods, by contrast, provide a more direct view of how a peptide may fit a defined site on the target protein and which residues are likely to contribute most strongly to the binding. Future workflows may therefore use sequence-based AI first to generate or prioritize candidates and remove peptides with likely off-target interactions, followed by structure-based analysis to refine residue placement and assess structural compatibility when suitable target information is available. In this way, the two strategies may function as complementary parts of a single design framework [260]. However, it is also important to recognize that sequence-based and structure-based approaches do not always identify the same candidates. This is partly because they are based on different assumptions about molecular recognition. A sequence-based model detects interaction patterns that are not obvious from a static 3D structure, while a structure-based model focuses on peptides that appear compatible with a specific binding site. As a result, the two approaches have the potential to point to alternative interactors rather than the same one. Having said that, when both methods independently support the same peptide–target interaction, confidence in that candidate may be higher.

AI-designed peptides may also serve as a starting point for the discovery of related natural peptides [249]. As discussed above, the same sequence-based logic used to generate artificial binders can also be applied in reverse to search natural proteomes for sequences with similar binding behavior. This raises an interesting possibility for cosmetic peptide discovery. AI could first be used to define or generate a peptide with the desired functional profile, after which natural sources could be mined for candidates with comparable properties. Such an approach may be especially attractive in the cosmetic field, where naturally occurring peptides may carry added consumer appeal. It also broadens the role of AI beyond de novo design alone and turns it into a tool for connecting artificial design with natural peptide discovery.

Future progress will also depend on the development of better datasets and models that are more closely tied to skin biology. Current AI approaches often rely on general peptide or protein interaction data, yet peptide behavior in cosmetic settings is strongly influenced by biological context. A peptide that performs well in a simplified predictive framework may not behave the same way in the skin, where issues such as local environment, target accessibility, stability, and unintended interactions all matter. For this reason, one important future direction is the creation of skin-relevant datasets and more context-aware models trained on properties that matter for cosmetic use. It is expected that improvements of this kind will help AI predictions to become more reliable and useful for the discovery of cosmetic peptides.

Lastly, future AI approaches will likely move beyond optimizing target binding alone and increasingly address several properties at the same time. In cosmetic applications, binding alone does not make a peptide useful. A candidate may bind its target well yet still be limited by functionality (for example, the ability to regulate enzymatic activity), poor stability, unfavorable toxicity, or weak performance in the relevant biological or formulation setting. For that reason, peptide design in this area should not focus on target recognition only. This is important since cosmetic peptides are not meant to function under idealized conditions alone. They are expected to perform within more complex environments, where factors such as stability, selectivity, safety, and formulation compatibility can all influence the suitability of a candidate as a meaningful cosmetic peptide. A peptide that looks promising at the level of binding may fall short if other features are not considered early in the design process. Multi-property design in parallel can potentially evaluate binding, selectivity, stability, solubility, toxicity, and delivery during the early stages of design. Such an approach could improve prioritization and reduce the number of peptides that appear promising at first but fail later for more practical reasons.

A practical next step for AI-assisted cosmetic peptide discovery may rely on building a clearer path from computational prediction to a useful cosmetic ingredient. This could begin with choosing a relevant and reachable skin target, followed by peptide generation and screening for binding and selectivity. Promising candidates would then need experimental testing for activity, stability, toxicity, skin penetration, and formulation compatibility. Clinical testing would come later, once these basic requirements are met. Considering these issues earlier may help reduce the number of candidates that look promising computationally but fail during later development.

## Figures and Tables

**Table 1:** Summary of peptides discussed in this review. The table lists the peptides discussed here, their origin or discovery/design approach, proposed category according to the conventional classification, proposed molecular target or functional pathway, and proposed mechanism according to the framework presented in this review, whether clinical evidence has been reported, and the selected literature.

Peptide Name	Origin or Discovery/Design Approach	Proposed Category *	Proposed Molecular Target or Functional Pathway **	Proposed Mechanism ***	Clinical Evidence	Selected Literature
Palmitoyl pentapeptide-4 (Pal-KTTKS)	From type I procollagen and palmitic acid	a	Extracellular matrix (ECM)-associated biosynthetic pathways	1	Yes	[25,26]
Glycyl-L-histidyl-L-lysine (GHK)-Cu	From human plasma tripeptide	b	Copper-dependent processes/multiple ECM pathways	1	No	[27,28]
Acetyl hexapeptide-8 (Argireline^®^)	From Synaptosomal-associated protein of 25 kDa (SNAP-25)	c	Soluble N-ethylmaleimide-sensitive factor attachment protein receptor (SNARE) complex assembly/muscle contraction	3	No	[29,30]
Decapeptide-12	As tyrosinase inhibitor	d	Tyrosinase/hyperpigmentation	5	Yes	[31,32]
Palmitoyl tripeptide-1 (Pal-GHK)	From human plasma tripeptide and palmitic acid	a	Primarily associated with ECM-associated biosynthetic pathways	1	Yes	[33,34]
Palmitoyl tetrapeptide-7	From immunoglobulin G and palmitic acid	a	Interleukin-6 (IL-6) and cytokine regulation/matrix homeostasis	4	No	[34,35]
Tripeptide-10 citrulline (Decorinyl^®^)	From Decorin	a	Collagen fibrils/ECM architecture	1	No	[36,37]
Waglerin-1	From *Tropidolaemus wagleri* venom	c	Muscle-type nicotinic acetylcholine receptors/muscle contraction	3	No	[38,39]
Dipeptide diaminobutyroyl benzylamide diacetate (SYN^®^-AKE).	From Waglerin-1	c	Muscle-type nicotinic acetylcholine receptors/muscle contraction	3	No	[38,39]
WP5, LW5 and YY6	From marine-derived antioxidant peptide	a	Kelch-like ECH-associated protein 1 (Keap1)- Nuclear factor E2 -related factor 2 (Nrf2) association/photooxidative damage	4	No	[40,41]
RMNE1 and its derivative, DualPep-Shine	As cell-penetrating peptide	a	Melanin synthesis/pigmentation	5	No	[42]
Pentasodium tetracarboxymethyl palmitoyl dipeptide-12 (PTPD-12)	As autophagy activator	a	Promotes autophagic flux/melanin accumulation	5	Yes	[43]
Tyr-Gly-Asp-Glu-Tyr (YGDEY)	From tilapia skin gelatin hydrolysates	a	Matrix metalloproteinases (MMPs)/skin photoaging	4	No	[44]
Seahorse-derived antioxidant peptide 2 (SHP2)	From *Hippocampus abdominalis*	a	Keap1-Nrf2 and UVB-induced reactive oxygen species (ROS)/photoaging	4	No	[40]
P12-3A	From insulin-degrading enzyme binder	d	Insulin-induced collagen-related responses/collagen synthesis	2	No	[45]
P15-1	In phage-display libraries against hyaluronan	a	Hyaluronan-related signaling/inflammation and fibrosis	2	No	[46]
pep_RTE62G	From *Pisum sativum* proteome	a	Keratinocyte proliferation/ECM-associated biosynthetic pathways	1	Yes	[47]
IK-16-1	From human ß-defensins	a	Antibacterial/ingredient protection	5	No	[48]

* The four conventional classifications introduced in this review are: a. signal peptides; b. carrier peptides; c. neurotransmitter-inhibiting peptides; d. enzyme-inhibitor peptides. Peptides were assigned to the category that reflects their proposed classification. However, some peptides may exhibit multiple activities. ** Peptides were assigned to the section according to their primary proposed target molecules and functional pathways. Some peptides may have multiple or overlapping target molecules and functions. *** The five conventional molecular mechanism categories introduced in this review are: 1. regulation of extracellular matrix synthesis and dermal remodeling; 2. modulation of matrix degradation and repair-associated processes; 3. neuromodulatory mechanisms related to expression lines; 4. modulation of inflammation, oxidative stress, and related signaling; 5. effects on pigmentation and other specialized pathways. Peptides were assigned to the category that reflects their proposed mechanism of action. However, some peptides, possibly exhibiting multiple biological activities or participating in various pathways, may overlap across more than one category.

**Table 2:** Comparison of representative AI-assisted peptide design approaches discussed in this review. The table summarizes the general type of approach, required input, main advantages and limitations, the level of validation or relevance to cosmetic peptide development, and the selected literature. These methods were originally developed for broader therapeutic research but may be transferable to future applications in cosmetics and skin health.

Approach	Type	Main Input	Main Advantage	Main Limitation	Application	Selected Literature
RFdiffusion	Structure-based	3D target structure with binding information	De novo structural design	Requires structural information	Therapeutic protein design	[219]
DiffPepBuilder	Structure-based	3D target structure with binding information	Target-specific sequence and structure design	Requires structural and binding region information	Therapeutic peptide design	[203]
ESM-IF1 + AlphaFold2	Structure-based	3D target structure with interface information	Structural evaluation	Dependent on predicted structural accuracy	Diagnostic and therapeutic peptide design	[200]
InSiPS/eLIFE	Sequence-based	Target sequences with positive and negative PPI databases	Large sequence-space search/negative selection (potentially fewer false positives)	No 3D structural information output/relies on protein-protein interaction (PPI) databases	Diagnostic and therapeutic peptide design	[194]
PepMLM	Sequence-based	Target sequence with binding information	De novo linear design	Limited output of binding information	Therapeutic peptide design	[201]

## Data Availability

The original contributions presented in this manuscript are included in the article. Further inquiries can be directed to the corresponding author.

## Abbreviations

The following abbreviations are used in this manuscript:

α-MSH	α-melanocyte-stimulating hormone
AP-1	Activator protein 1
CREB	Element-binding protein
ECM	Extracellular matrix
HO-1	Heme oxygenase-1
IL-1	Interleukin-1
IL-6	Interleukin-6
IL-8	Interleukin-8
Keap 1	Kelch-like ECH-associated protein 1
L-DOPA	L-3,4-dihydroxyphenylalanine
MAPK	Mitogen-activated protein kinase pathways
MITF	Microphthalmia-associated transcription factor
MMPs	Matrix metalloproteinases
NF-κB	Nuclear factor kappa B
Nrf 2	Nuclear factor E2-related factor 2
Pal-GHK	Palmitoyl tripeptide-1
Pal-KTTKS	Palmitoyl pentapeptide-4
PPI	Protein–protein interaction
PTPD-12	Pentasodium tetracarboxymethyl palmitoyl dipeptide-12
RHAMM	Receptor for hyaluronan-mediated motility
ROS	Reactive oxygen species
SARS-CoV-2	Severe acute respiratory syndrome coronavirus 2
SHP2	Seahorse-derived antioxidant peptide 2
SNAP-25	Synaptosomal-associated protein of 25 kDa
SNARE complex	Soluble N-ethylmaleimide-sensitive factor attachment protein receptor complex
SPARC	Secreted protein acidic and rich in cysteine
TNF-α	Tumor necrosis factor-α
VAMP	Vesicle-associated membrane protein
